# Alphavirus Replication: The Role of Cardiac Glycosides and Ion Concentration in Host Cells

**DOI:** 10.1155/2020/2813253

**Published:** 2020-05-09

**Authors:** Kauê F. C. Souza-Souza, Cassiano F. Gonçalves-de-Albuquerque, Cláudio Cirne-Santos, Izabel C. N. P. Paixão, Patrícia Burth

**Affiliations:** ^1^Departamento de Biologia Celular e Molecular, Instituto de Biologia, Universidade Federal Fluminense, Niterói, RJ CEP 24020-141, Brazil; ^2^Departamento de Bioquímica, Universidade Federal do Estado do Rio de Janeiro, Rio de Janeiro, RJ CEP 20211-010, Brazil; ^3^Laboratório de Imunofarmacologia, Instituto Oswaldo Cruz, FIOCRUZ, Rio de Janeiro, RJ CEP 21040-900, Brazil

## Abstract

Alphaviruses are arthropod-borne viruses that can cause fever, rash, arthralgias, and encephalitis. The mosquito species *Aedes aegypti* and *Aedes albopictus* are the most frequent transmitters of alphaviruses. There are no effective vaccines or specific antivirals available for the treatment of alphavirus-related infections. Interestingly, changes in ion concentration in host cells have been characterized as critical regulators of the alphavirus life cycle, including fusion with the host cell, glycoprotein trafficking, genome translation, and viral budding. Cardiac glycosides, which are classical inhibitors of the Na^+^ K^+^ ATPase (NKA), can inhibit alphavirus replication although their mechanisms of action are poorly understood. Nonetheless, results from multiple studies suggest that inhibition of NKA may be a suitable strategy for the development of alphavirus-specific antiviral treatments. This review is aimed at exploring the role of changes in ion concentration during alphavirus replication and at considering the possibility of NKA as a potential therapeutic target for antiviral drugs.

## 1. Introduction

Viruses of the genus *Alphavirus* genus are a subset of the family Togaviridae [1]. Among the 31 virus species that are included in this family [2], only salmon pancreatic disease virus and Southern elephant seal virus are not arthropod-borne [3]. The *Alphavirus* genus includes Eastern equine encephalitis virus, Venezuelan equine encephalitis virus, and Western equine encephalitis virus, which are pathogens that can infect mammalian species and cause encephalitis [4]. Other members of this genus include Chikungunya virus (CHIKV), O'nyong-nyong virus, Ross River virus, Semliki Forest virus, Mayaro, and Sindbis virus; infections with these viruses are associated with fever, rash, and arthralgias [5]. Alphavirus virions are small, regularly-shaped spherical particles with positive-sense single-stranded RNA genome covered by an icosahedral capsid (nucleocapsid) that contains glycoprotein components in an icosahedral lattice [6]. The capsid consists of two icosahedral shells that are formed from a host-derived membrane bilayer [7] located between the inner and outer shells and penetrated by transmembrane domain anchors of E1 and E2 proteins [8]. The E2 domain is essential for maintaining interactions with E1 and the capsid protein and is a critical target of neutralizing antibodies [9].

The primary vectors responsible for alphavirus infections are the mosquitos *Aedes aegypti* and *Aedes albopictus* [10]. Uncontrolled urbanization favors vector expansion, boosts the emergence of viruses, and interferes with infection control measures [11]. Currently, there are no effective vaccines or treatments for disease caused by these pathogens [12]. An alternative approach might include antiviral drugs that target critical host proteins, similar to what has been done for human immunodeficiency virus [13, 14]; however, at this time, the role of host proteins in the virus lifecycle has not been studied to a sufficient degree [7, 15].

Decades ago, several reports documented changes in ion concentrations within host cells that were linked to viral replication [16]. For example, increasing the NaCl concentration in tissue culture medium directly inhibits maturation and release of the Sindbis virus, Semliki Forest, and vesicular Stomatitis virus [17]. By contrast, elevated NaCl concentrations were also associated with increased transcription efficiency of Sindbis virus messenger RNA (mRNA) [18]. The significance of the Na^+^ ion concentration and its impact on reducing viral yield was also considered in experiments focused on Chikungunya virus (CHIKV) infection in human osteosarcoma cells. Interestingly, treatment of human cells with digoxin or the related cardiac glycoside, ouabain, resulted in a dose-dependent decrease in the efficacy of CHIKV infection. Other alphaviruses, including Ross River virus and Sindbis virus, as well as mammalian reovirus and vesicular stomatitis virus, are sensitive to the antiviral activity of digoxin [19].

In 2015, Fields and Kielian documented the critical role of H^+^ ion concentration in the mechanism underlying alphavirus fusion [20]. Increased H^+^ ion concentration was also required for nucleocapsid disassembly and translocation of the viral genome [21]. Therefore, a more in-depth analysis of proteins that regulate the ion flow within host cells, notably the aforementioned Na^+^ K^+^ ATPase (NKA), may reveal new targets and therapeutic strategies for the treatment of alphavirus infections.

## 2. Na^+^ K^+^ ATPase (NKA)

NKA is a transmembrane enzyme. Its mechanism of action was explored many years ago and includes its capacity for ion exchange, specifically the transfer of three Na^+^ ions to the extracellular space in exchange for two K^+^ ions imported into the cell cytosol, accompanied by the hydrolysis of ATP. NKA activity is crucial for maintaining the electrochemical gradient and cellular osmolarity [22]. Appropriate NKA function is critical factor for renal filtration, reabsorption of amino acids and glucose, and regulation of electrolyte and pH levels in the blood [23] as well as sperm motility and generation of neuronal action potentials [24]. NKA includes three submits known as *α*, *β*, and *γ* [24]. The *α* catalytic subunit contains binding sites for Na^+^, K^+^, and Mg^++^ ions, ATP, and cardiac glycoside inhibitors [25]. The *β* subunit stabilizes and guides *α* subunit within the membrane and controls its affinity for K^+^ ions and cardiac glycoside inhibitors [26]. The *γ* subunit modulates the affinity for Na^+^ and K^+^ ions [24]. NKA can also transduce signals from the extracellular space [27]. This complex, multisubunit function may have been acquired during the evolution by incorporation of many domains that interact with specific ligands and intracellular proteins, similar to what has been observed for the protooncogene tyrosine-protein kinase Src (Src), protein kinase C (PKC), phosphoinositide 3-kinase (PI3K), protein kinase A (PKA), extracellular signal-regulated kinase (ERK), and the caveolins [28–30]. This function, however, seems to be restricted to caveolae and unrelated to the Na^+^ K^+^ exchange function [31, 32].

Cardiac glycosides are classical inhibitors of the NKA [30]. They are used in the treatment of patients with heart failure because of their potent inotropic effect [33, 34]. Cardiac glycosides, such as ouabain and digoxin, disrupt the flow of the Na^+^ and K^+^ ions, resulting in a significant change in the cellular ion gradient. Inhibition of NKA generates an increase in intracellular Na^+^ concentration and activates Na^+^/Ca^++^ exchanger; this results in an increase in intracellular Ca^++^ which promotes cardiac contractility [33, 35]. Cardiac glycosides also trigger signal transduction and can activate intracellular pathways through NKA at low concentrations [30, 36].

The cardiac glycosides have also been linked to antiviral activity [37–40]. For example, adenovirus relies on the host pre-RNA splicing machinery for genome expression; as such, this virus will be vulnerable to digoxin and digitoxin, which can modulate RNA splicing. The yields of human adenovirus are reduced by at least 2 to 3 logs by these drugs. Cardiac glycosides can disrupt virus genome replication and transcription but do not promote target cell death [41]. Furthermore, Cai et al. [42] reported that cardiac glycosides inhibit cytomegalovirus replication and that the combination of ganciclovir (GCV) and cardiac glycosides (digoxin, digitoxin, and ouabain) resulted in additive antiviral effects. Despite these impressive effects, there are comparatively few studies that directly address the role of cardiac glycosides as putative antiviral agents [43].

## 3. Influence of the Ion Change during Attachment, Fusion, and Nucleocapsid Disassembly

*Alphavirus* infection is initiated by binding to the surface cell host receptor [44] which results in fusion and internalization [45]; an alternate pathway for cell penetration includes direct transfer of virus RNA through pores formed in the plasma membrane during infection [46–48]. As part of the fusion process, the E2 envelope protein binds to cell receptors [49], and the E1 protein acts to promote viral envelope fusion with the host endosomal membrane [50]. The E3 protein protects against premature activation of E1 and facilitates pE2/E1 heterodimerization [51]. Interestingly, the viral particles inhibit NKA during attachment to the host cell membrane [52]; Carrasco [16] reported that infection with Semliki Forest altered ion gradient at the host cell membrane.

During virus fusion with the host cell membrane, the high H^+^ concentration inside the endosome promotes capsid protein degradation [47]. The degradation occurs because of high H^+^ concentrations which result in E1 protein permeability to Na^+^, K^+^, and Ca^+^ ions which allows endosomal protons to flow into the cytoplasm via exchange K^+^ ions. The high H^+^ ion concentration within the endosomes promotes nucleocapsid disassembly and translocation of the viral genome [21]. The cellular 60S ribosome efficiently removes the nucleocapsid proteins and exposes the viral RNA [53, 54]. An alternate mechanism for nucleocapsid disassembly involves pore formation by the virus E1 protein which serves to direct protons into the viral particle, thereby facilitating the uncoating viral RNA [55] via the actions of ion channels that promote Na^+^/Cl^+^ and Na^+^/K^+^ exchange by the viral protein 6 k [56].

## 4. Role of Ion Change in Viral Genome Replication

*Alphavirus* genome is a single strand of positive sense RNA; coding segments at the 5′ end of the genome are those related to genomic replication and mRNA synthesis, and coding sequences at the 3′ end are those that encode virus structural proteins [6, 57]. Nonstructural proteins, including nsP1 (methyltransferase), nsP2 (helicase), nsP3 (an accessory in the synthesis of the negative RNA strand), and nsP4 (RNA polymerase), are generated by the sequential cleavage of the nonstructural polyprotein P1234 [58, 59]; these proteins are components of the replication complex within cytopathic vacuoles [60]. The functional arrangement within the replicase complex is not clearly understood [15]. The structural proteins, including C (capsid protein), E1 (glycoprotein), pE2 (which is cleaved in the Golgi to generate E2 and E3 glycoproteins), and a small peptide termed 6 K, are all parts of a cation-selective ion channel [56, 61] and are translated from a subgenomic mRNAs as a polyprotein (p130) on the polysomes [62]. C protein, the first to be synthesized, is responsible for carrying out self-cleavage and for releasing capsid protein into the cytoplasm [62]. In the cytoplasm, the C protein includes a signal sequence directing it to the endoplasmic reticulum, where it undergoes further processing to become a viral capsid protein [62].

The nonstructural proteins have been identified as attractive targets for antiviral therapeutics [63, 64]. Digoxin induces mutations in non-structural proteins, including nsP4, in CHIKV-infected cells, resulting in disruption of RNA synthesis and/or the replication complex that is essential for alphavirus replication [19].

Bafilomycin A1 (BAF) is a specific inhibitor of the vacuolar-ATPase (V-ATPase) which is responsible for the acidification of endosomes. BAF has been used to inhibit alphavirus infection, with the assumption that the acidification of endosomes by the V-ATPase was required for penetration, endocytosis, and intracellular vesicle transport. A functional V-ATPase is required for efficient alphavirus RNA synthesis and maturation [65].

Increasing NaCl concentration in the culture medium of epithelial osteosarcoma cells treated with digoxin (0.25 *μ*M) resulted in significant inhibition of CHIKV yield; increasing the KCl concentration had the opposite effect [19]. Increases in the intracellular concentrations of Na^+^ in cells infected by Sindbis virus resulted in inhibited protein synthesis and increases in the levels of untranslated mRNA in the host cells; these studies suggest a crucial role for Na^+^ with respect to virus replication [66]. Taken together, these results suggest that digoxin actions on viral replication via its ability to inhibit NKA [19].

## 5. Influence of the Ion Change during Morphogenesis and Viral Budding

Viral morphogenesis begins just after the replication process. The newly synthesized capsid proteins are transferred directly to the nucleocapsid or released in association with 80S monosomes [67]. The region that includes amino acids 81 to 113 of the Sindbis virus capsid protein is responsible for encapsidation of the viral RNA and for the accumulation of nucleocapsid proteins in the cytoplasm of infected cells [68].

The assembly of viral structural proteins at the plasma membrane prior to the release of viral particles is linked to the inhibition of NKA [69]. The entry of alphaviruses into the plasma membrane converts proteins from the viral surface into an ion-permeable pore. Available evidence suggests that the alphavirus E1 protein probably forms these pores [53]. The 6 k protein is a small polypeptide that is rich in cysteines that is typically associated with p62-E1 heterodimers (p62 is the precursor of E3 and E2) [70] and interacts with fatty acids inserted on a lipid bilayer via a hydrophobic sequence [71]. The activities of 6 k are crucial for the successful release of virions from host cells [72]. In addition to its role in creating an ion channel that is selective for Na^+^, K^+^, Ca^++^, and Cl^−^ ions, the 6 k protein participates in glycoprotein trafficking [73] and can induce caspase-dependent programmed cell death [74]. The 6 k protein functions as an ion channel during virus budding [56, 75].

## 6. Na^+^ K^+^ ATPase as a Potential Drug Target

Cardiac glycosides have antiviral activity in the setting of alphavirus infection [19, 76]. Ganesan et al. [77] showed that ouabain exerted a specific inhibitory effect on Sindbis virus RNA synthesis in *Aedes albopictus* cell lines that were resistant to the gene silencer, bromodeoxyuridine [77]. The antiviral activity of cardiac glycoside includes interference prior to virus attachment, as has been shown in human osteosarcoma cells (U-2 OS) infected with CHIKV virus, River virus, Sindbis virus, and vesicular stomatitis virus [19]. Membrane fractions obtained from Sindbis virus-infected chick embryo cells also were also found to be ouabain-sensitive [78], as were other alphavirus pathogens [41, 79–83].

As the NKA is the sole target of cardiac glycosides, the aforementioned results strongly suggest a link between the antiviral effect of these agents and NKA function [19]. The cardiac glycoside mechanism of antiviral action, however, remains unclear. We suggest two possible hypotheses: first, cardiac glycosides have a clear impact on intracellular ion concentrations, and second, they trigger intracellular signaling cascades that can impair virus replication. Some data suggest that Src activation by cardiac glycosides can trigger MAPK (extracellular signal-regulated kinase (ERK) 1/2) signaling through the Raf-MEK cascade, resulting in the inhibition of viral gene expression; however, these data were generated in HIV-infected cells treated with a cardiotonic steroid [84].

Overall, cardiac glycosides create an unfavorable environment for virus replication [36]. The possibility that the cardiac glycoside/NKA axis could be targeted by novel antiviral therapeutics merits further consideration.

## 7. Conclusions

**Figure 1:**
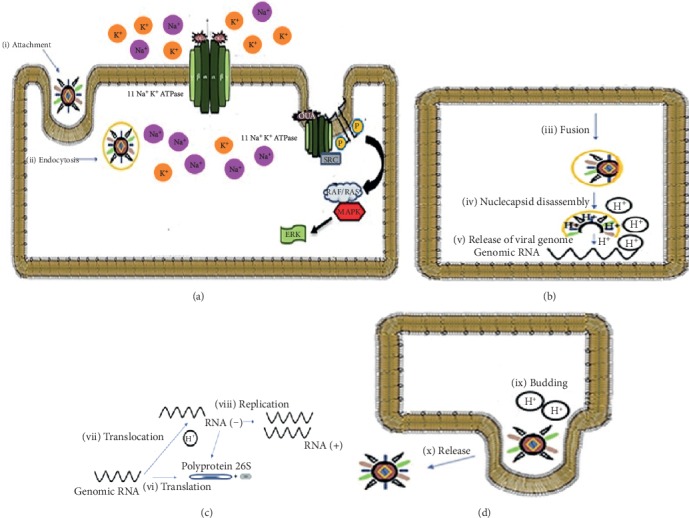
The role of ion concentration during alphavirus replication. Alphavirus infection is initiated by attachment (a-i) and binding to the host cell surface receptor and the virus is then internalized by endocytosis (a-ii). During virus fusion with the host cell membrane (a-iii), a high H^+^ concentration inside the endosome promotes degradation of the capsid protein, nucleocapsid disassembly (b-iv), and release of the viral genome into the cytoplasm (b-v). The genomic RNA is the template for protein translation from both whole genomic and subgenomic (26S) RNA (c-vi) and also the transcription of RNA (+) via a RNA (−) template intermediary (c-vii). The viral genome is replicated (c-iii) and incorporated into a new viral particle. The last phase is the virus budding at the host cell membrane in the presence of elevated H^+^ ion concentration (d-ix) followed by virion release (d-x). The inhibition of Na^+^ K^+^ ATPase by the cardiac glycoside, ouabain (OUA) (a-xi), results in a change cell osmotic concentration and (c-vi) interrupting synthesis polyprotein through ion change and/or by cell signaling pathways that limit virus replication.

The alphavirus replication cycle is profoundly affected by changes in host intracellular ion concentrations (Figure 1). Changes in the concentration of the Na^+^ and K^+^ ions interfere in the gene expression, virus yield, and can inhibit intracellular viral protein synthesis. The inhibition of CHIKV replication by the cardiac glycoside, digoxin, can be altered by increasing the extracellular potassium concentration, suggesting that antagonism of sodium-potassium ATPase is a critical mediator of the antiviral effect. The antiviral effect may be directly associated with NKA inhibition [19, 37], suggesting that this enzyme may be a target for novel antiviral therapeutics. We believe that further studies will unveil the precise mechanisms of action of antiviral drugs targeting NKA and will boost further research in the field.
